# Microelectronic Structure and Doping Nonuniformity of Phosphorus-Doped CdSeTe Solar Cells

**DOI:** 10.1021/acsami.4c15741

**Published:** 2025-01-06

**Authors:** Chun-Sheng Jiang, Rouin Farshchi, Timothy Nagle, Dingyuan Lu, Gang Xiong, Lorelle M. Mansfield, Matthew O. Reese

**Affiliations:** † National Renewable Energy Laboratory, Golden, Colorado 80401, United States; ‡ California Technology Center, First Solar Inc, Santa Clara, California 95050, United States

**Keywords:** CdTe solar cell, phosphorus doping, microelectronic structure, Kelvin probe force microscopy (KPFM), scanning spreading resistance microscopy (SSRM)

## Abstract

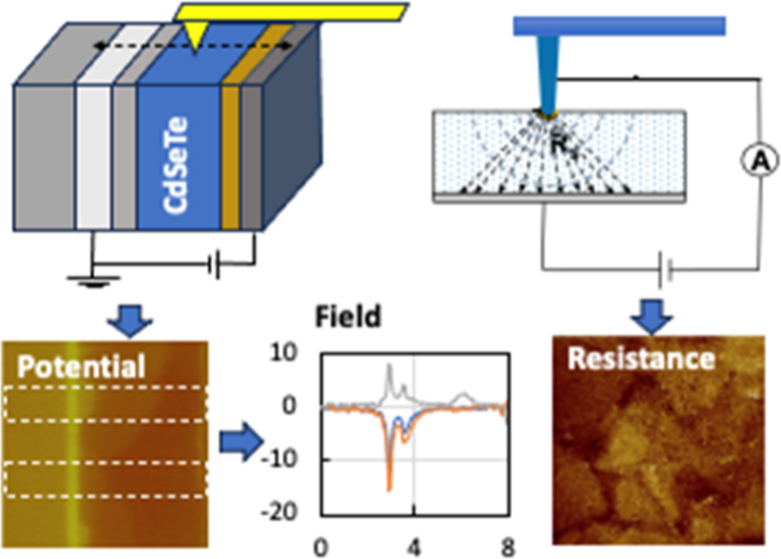

Optimizing group-V doping and Se alloying are two main focuses for advancing CdTe photovoltaic technology. We report on nanometer-scale characterizations of microelectronic structures of phosphorus (P)-doped CdSeTe devices using a combination of two atomic force microscopy-based techniques, namely, Kelvin probe force microscopy (KPFM) and scanning spreading resistance microscopy (SSRM). KPFM on device cross-section images distribution of the potential drop across the device. SSRM taken on a delaminated front interface and further beveling into absorber bulk reveals local distributions of doping polarity and carrier concentration. The KPFM and SSRM imaging corroborate each other, suggesting that nonuniform doping revealed by SSRM is associated with nonuniform potential features observed by KPFM. These detrimental microelectronic structures were improved by enhancing P-doping. The large nonuniform potential drop and deep overall n–p transition in the device without doping were mitigated to potential fluctuation around the front interface and n–p transition depth of ∼100 nm by low-level P-doping and further mitigated to scarce and slight irregular potential and p-weighed doping at the interface by high-level P-doping. These characterizations imply sophisticated defect chemistry, atomic structure, and associated electronic structure in CdTe with Se alloying and group-V doping together and further point to the direction for improving device efficiency by mitigating and ultimately eliminating the nonuniform doping and irregular potential.

## 1 Introduction

CdTe-based thin film solar cell is one of the mainstream photovoltaic (PV) technologies dominating the current PV market. In addition to the wide deployment in utility-scale solar farms, the monolithic structure of CdTe PV modules enables uniquely building-integrated PV and various applications with flexible substrates. Currently, optimizing group-V (Gr-V) doping and Se incorporation are two main focuses for advancing the CdTe technology. The recent record efficiency device is implemented with improvements on both fronts.^1^ Gr-V doping has effectively improved the carrier concentration from 10^14^/cm^3^ from traditional Cu-doping to 10^16^/cm^3^ that leads to increased quasi-Fermi-level splitting and improvement of open-circuit voltage (*V*_oc_) exceeding 1 V in single-crystalline devices.^2−6^ Se incorporation is believed to passivate nonradiative recombination defects in the absorber and further induces conduction band grading that leads to enhanced electron transport toward the emitter.^7−10^ However, *V*_oc_ of As-doped polycrystalline devices has been limited to <920 mV so far.^1^ Arsenic (As) and its oxide segregation at the front interface and nearby have been observed.^2,11^ The activation rate of Gr-V doping in the CdSeTe thin films was widely observed to be limited to a few percent. The absorber defect chemistry can vary significantly according to the choice of the dopant, degree of Se alloying, and their combinations.^12^ The defect chemistry, atomic structure, and associated electronic structure not only change common properties such as the absorber minority carrier lifetime and its passivation strategy but can also change the microelectronic structure of the devices, such as the local doping type and carrier nonuniformity on the microscale. Electronic characterizations with nanometer resolution are expected to tackle these issues in the current R&D efforts.

In this contribution, we report characterizations on phosphorus (P)-doped CdSeTe devices using a combination of two atomic force microscopy (AFM)-based nanoelectrical probes, namely Kelvin probe force microscopy (KPFM)^13,14^ and scanning spreading resistance microscopy (SSRM).^15,16^ KPFM on the device cross sections measures the distribution of potential drop across the device. SSRM taken on a delaminated front interface and further beveling into the absorber bulk reveals the local doping type and carrier distribution. These techniques can directly tackle nanometer-scale nonuniform doping and potential distributions that are detrimental to the device performance. The KPFM and SSRM characterization results illustrate nonuniform potential drops and an apparent highly resistive thin layer that can be associated with local low- and nonuniform doping near the front interface. These detrimental features for device performance are significantly improved by enhancing P-doping.

## 2 Results and Discussion

CdSe_*x*_Te_1–*x*_ absorbers were deposited using vapor transport deposition (VTD) at a thickness of 4–5 μm on glass substrates coated with fluorine-doped tin oxide as the transparent conductive oxide (TCO). The CdSe_*x*_Te_1–*x*_ layers have graded Se content such that in the finished devices *x* values are ∼20–30% at the front of the absorber with band gap *E*_g_ ∼1.4 eV (near the TCO) and below 5% at the back with *E*_g_ ∼1.5 eV. Phosphorus is incorporated after the VTD step into the CdSeTe absorber, followed by a chlorine heat treatment (CHT) step where annealing is carried out in the presence of CdCl_2_ at temperatures between 400 and 500 °C. During the CHT step, P activation occurs in addition to Se diffusion, chlorination, and grain growth. It should be noted that any nonuniformities in the reactions occurring during CHT are expected to be the main factors driving spatial nonuniformities observed in the results presented below. The devices are finished by the deposition of a thin ZnTe layer, followed by a metal stack.

Three devices with different P-doping levels are characterized using KPFM potential imaging and SSRM resistance imaging. Phosphorus was introduced into the absorber layer at: (i) “no P”, (ii) “low-P” with ∼3 × 10^17^ cm^–3^ uniform concentration in the finished absorber, and (iii) “high-P” with ∼1 × 10^18^ cm^–3^ uniform concentration in the finished absorber. The polycrystalline film is in the zinc blende phase in the finished devices. Device performances of the three devices are listed in Table 1.

**Table 1:** Device *I*–*V* Performance for the Finished Devices

condition	efficiency [ %]	*V*_oc_ [mV]	*J*_sc_ [mA/cm^2^]	FF [%]
no P	3.8	554	9.2	70.75
Low-P	15.9	772	28.7	71.74
High-P	17.2	816	29.0	72.72

### 2.1 KPFM Potential Imaging across Devices

**Figure 1:**
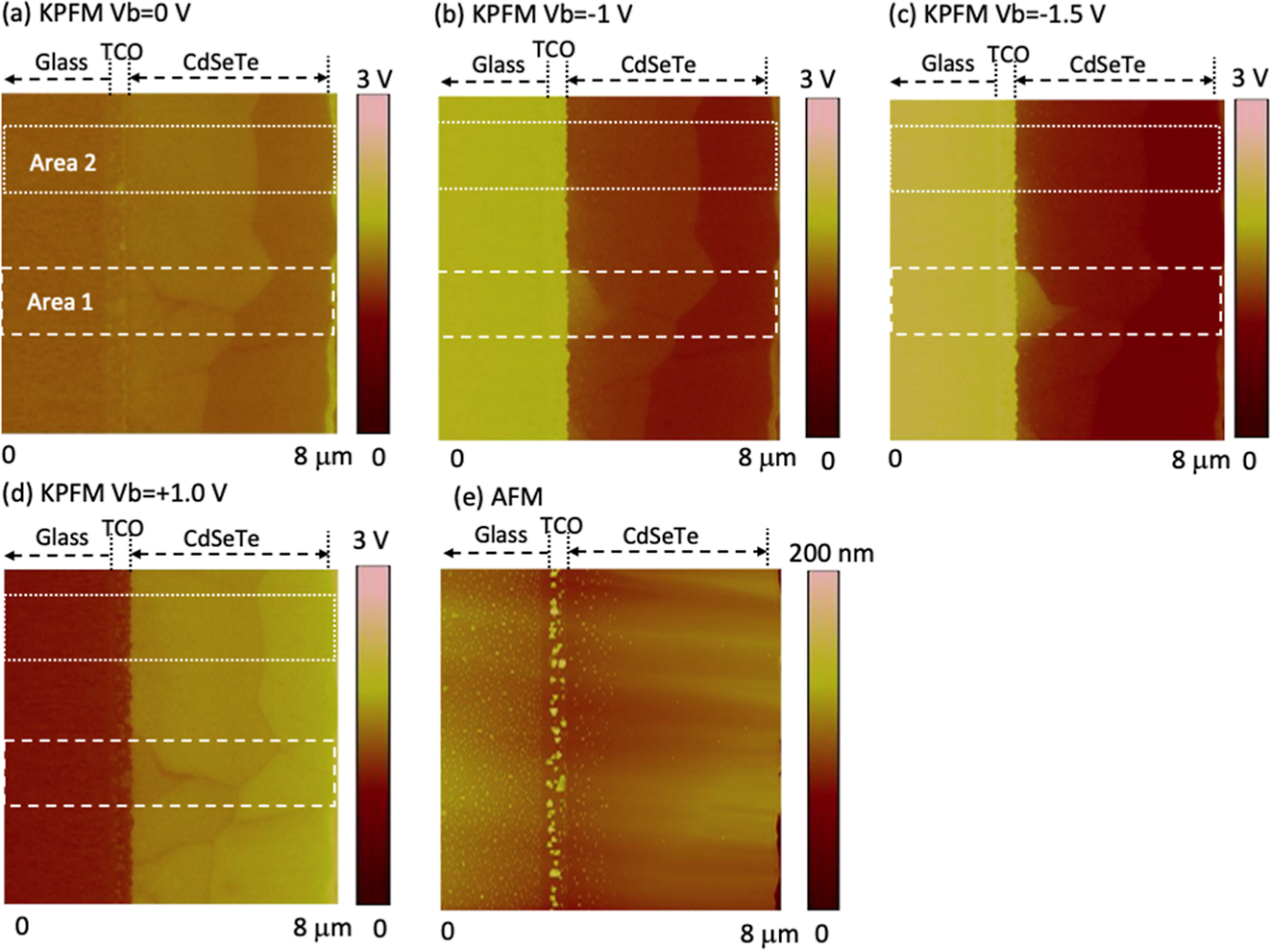
(a–d) KPFM images taken on the cross section of a high-P device on the same area under different bias voltages *V*_b_ and the (e) corresponding AFM image taken simultaneously with the KPFM images. Areas 1 and 2 denote regions with different potential distributions, profiles of which are shown in Figure 2.

KPFM images surface potentials in ∼30 nm spatial and ∼10 mV voltage resolutions. Figure 1 shows KPFM images taken on the cross section of the device with a high-level P-doping (high-P). A schematic of the device structure and KPFM imaging is shown in Figure S1. A bias voltage (*V*_b_) is applied to the device with the TCO grounded and *V*_b_ applied to the back contact. Four images were taken on the same area, with *V*_b_ = 0 V, reverse bias *V*_b_ = −1, −1.5 V, and forward bias *V*_b_ = +1 V, to examine the potential drops by the reverse and forward *V*_b_ (Figure 1). The potential changes with the different *V*_b_ were observed mostly at the TCO/CdSeTe interface (such as area 2 in Figure 1), except for a small area of area 1 where a weak potential drop was also observed at a distance of ∼750 nm from the front interface in addition to the dominant potential drop at the interface. In addition to the grain boundary (GB) perpendicular to the film plane, GBs or layer boundary were also observed in the direction parallel to the film plane in a vertical distance roughly in the middle of the film. A visible potential drop can be seen with forward *V*_b_ = +1 V, indicating a potential barrier at the lateral GBs for carrier transport. Different from these lateral GBs, no *V*_b_-induced voltage drop was observed across the vertical GBs among the grains grown in the film’s vertical direction. A weak potential contrast between the GB and the grain interior (GI) shows a slightly lower potential at the GBs. However, this potential contrast on the surface depends on cross-sectional sample preparation and does not reflect GB potential in the film bulk.

**Figure 2:**
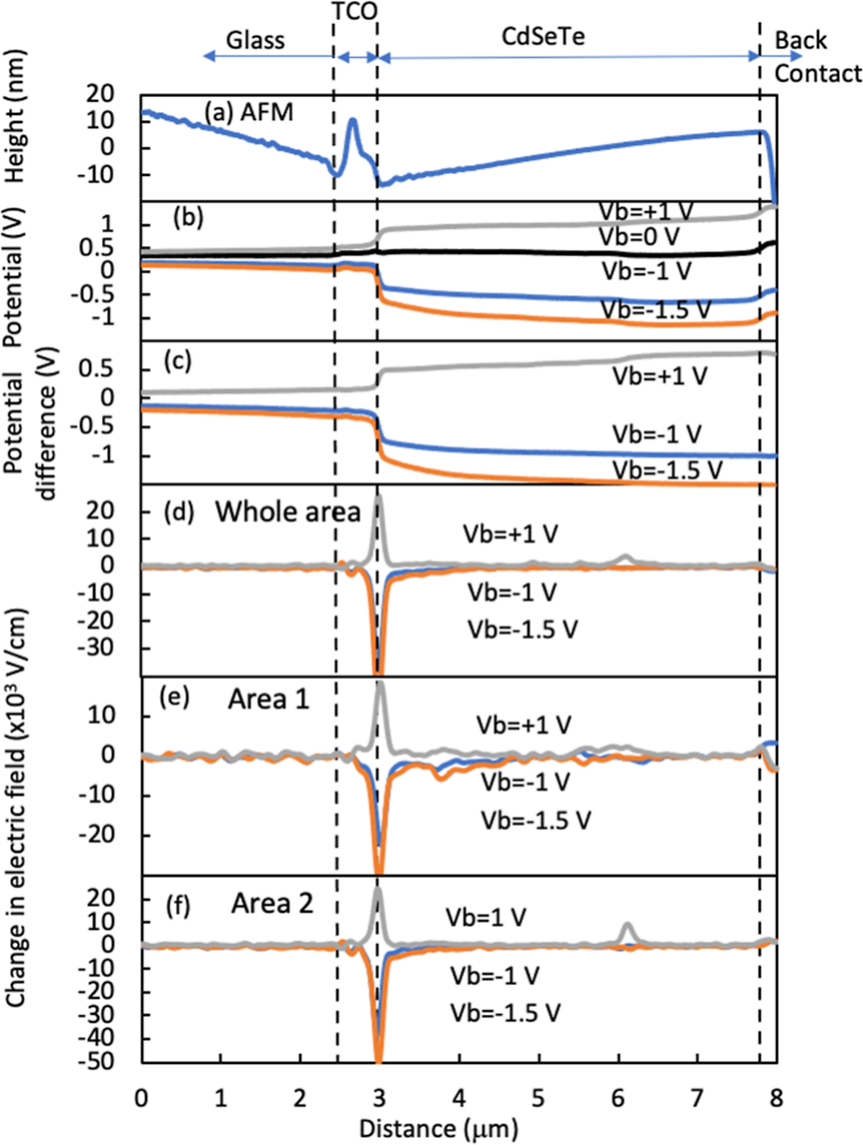
(a–d) Profiles of AFM, potential, potential difference, and electric field difference averaged from the whole scan area in Figure 1. (e,f) Averaged electric field from the areas indicated in Figure 1.

To exhibit the potentials in more detail, the potential profiles averaged from the images along the direction parallel to the device are shown in Figure 2b. This average is necessary to enhance the signal/noise ratio when deducing the electric field in the film vertical direction (*x*-direction) by taking a derivative of d*V*/d*x*, as discussed later. The averaged AFM profile (Figure 2a) is used to identify the boundary locations between the TCO, absorber, and back contact. The surface potential at *V*_b_ = 0 V (Figures 1a and 2b) exhibits a rather flat potential across the device instead of the built-in potential across the p–n junction, due to the charges trapped at the cross-sectional surface states (see Figure S2).^17,18^ To probe the potential in the device bulk, the surface potential is measured with applying a bias voltage, *V*_b_ (Figure 2b), and the potential change in the bulk by *V*_b_ is approximately identical to the measured surface potential change (Figures 2c and S2), as the surface charges are localized at the surface states and do not drift with a small *V*_b_ < 2 V.^17,18^ Two reverse *V*_b_ voltages were selected for the potential imaging that makes the surface potential from the nearly flat condition toward the direction of built-in potential in the bulk (Figure S2). One forward *V*_b_ voltage was selected that can examine the voltage drop across the barriers for current flow. The corresponding electric field changes induced by *V*_b_ are further deduced by taking the derivatives of the potential changes d*V*/d*x* (Figure 2d). The electric field change by applying *V*_b_ is in the *x*-direction, as the *V*_b_ is applied across the device. In this way, we can deduce the *V*_b_-induced potential drop and electric field change across the device, by avoiding effects from the surface charges on cross sections. However, the built-in potential across the device at *V*_b_ = 0 V and the absolute potential distribution in the bulk under *V*_b_ applied could not be measured by KPFM. Additional descriptions of the data processing and rationality can be found in the Supporting Information.

Overall, the *V*_b_-induced electric field concentrates at the front junction (Figure 2d), representing a relatively healthy electronic structure of the device, as expected from a healthy device. However, a small area (area 1 in Figure 1) shows an odd potential distribution where a potential drop or a secondary electric field peak is observed at a distance of ∼750 nm from the front interface (Figure 2e). This electric field profile suggests a possible local n–i–p structure with a very low-doped region of ∼750 nm wide or a shift of the local p–n junction from the interface to the peak location.^17^ In contrast, only one sharp electric field was measured in most areas (Figure 2f) with an extension of the electric field of less than 500 nm. The small odd region makes the averaged overall electric field appear with a long tail into the bulk (Figure 2d). The potential drop under forward *V*_b_ = +1 V across lateral GBs is represented as the peak at a distance of ∼3 μm from the interface, illustrating a moderate potential barrier for carrier transport in forward *V*_b_.

**Figure 3:**
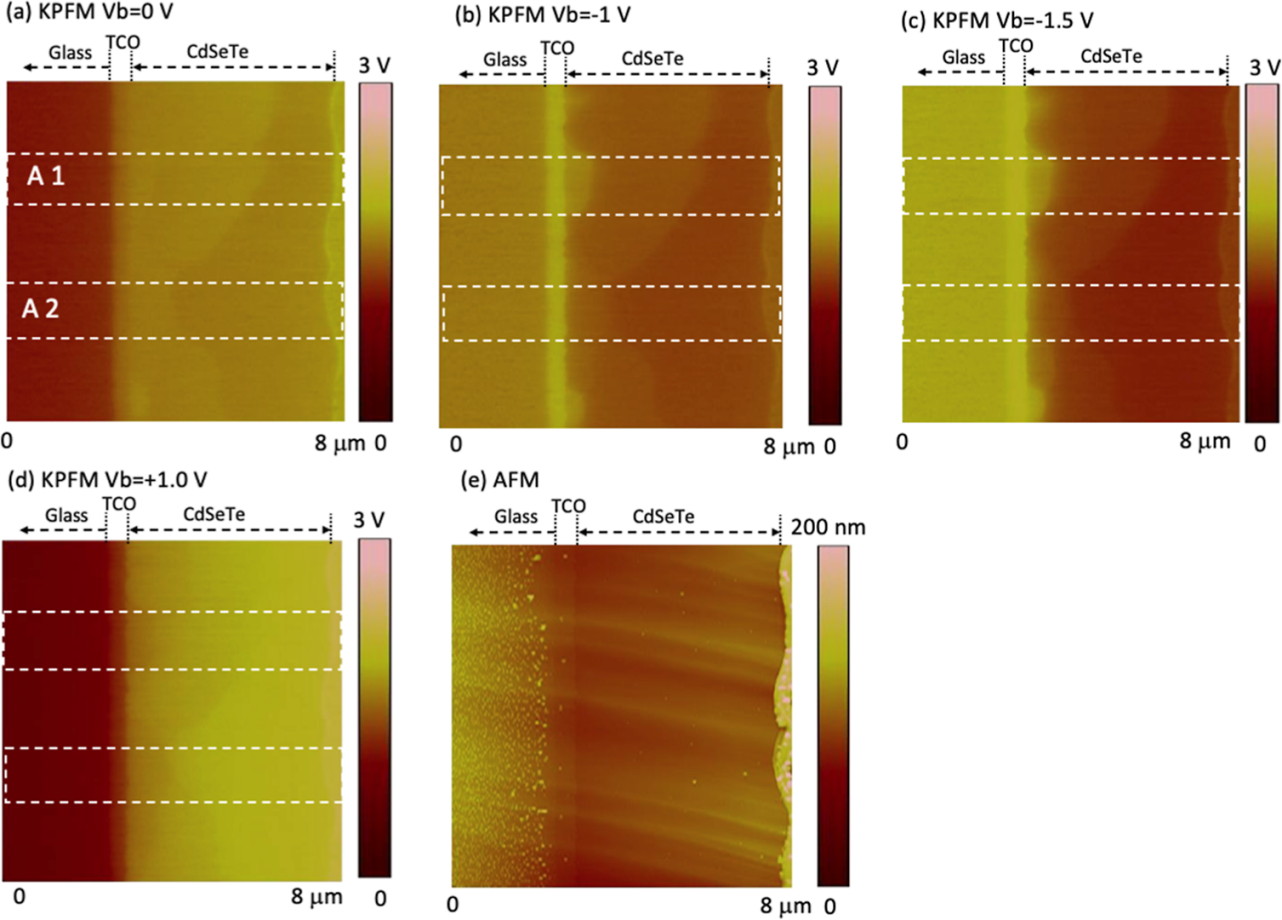
(a–d) KPFM images taken on the cross section of low-P device on the same area under different bias voltages *V*_b_ and (e) corresponding AFM image taken simultaneously with the KPFM images. Areas 1 and 2 denote regions with different potential distributions, profiles of which are shown in Figure 4.

**Figure 4:**
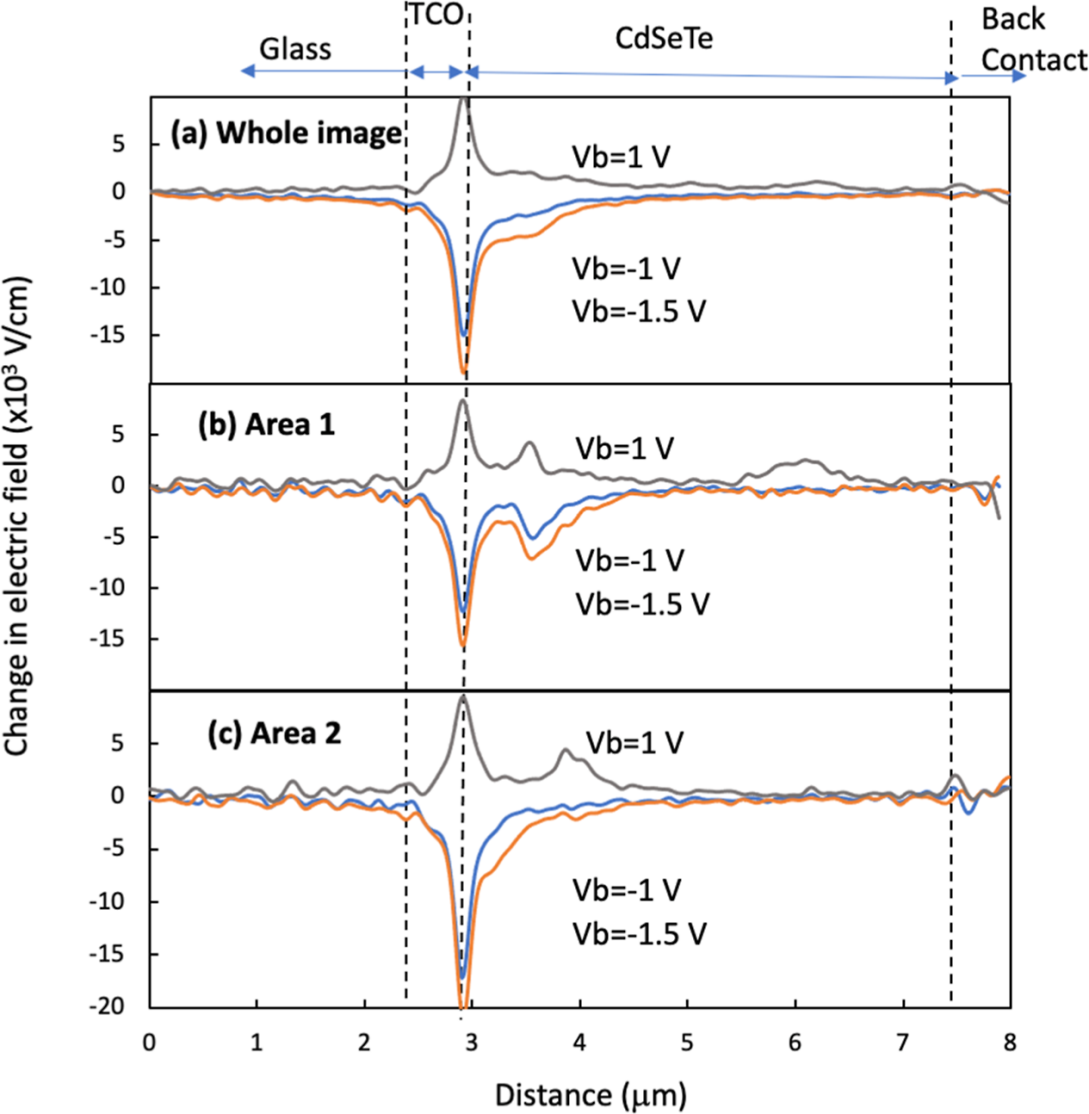
Change in the electric field induced by *V*_b_ across the low-P device, averaged over (a) whole image, (b) area 1, and (c) area 2, as indicated in Figure 3.

Figures 3 and 4 show the potential images and the profiles of *V*_b_-induced electric field taken on the device with low-level P-doping (low-P) under different *V*_b_. The profiles of AFM, potential, and potential changes, together with the electric field changes, are shown in Figure S3. Overall, the potential distribution around the junction area is more inhomogeneous than that for the high-P device, illustrating a more nonuniform junction structure. The potential drop is frequently extended deep into the absorber bulk, indicating the local n–i–p or n–p structure in the CdSeTe bulk. Similar to the high-P device, there are lateral GBs in the CdSeTe bulk parallel to the device plane at depths further away from the front interface than the extension of the potential drop (Figure 3). The electric field in area 1 (Figure 4b) exhibits an electric field peak at a location ∼750 nm from the interface with both forward and reverse *V*_b_, corresponding to the bump in the potential images (Figure 3), indicating a buried n–p junction. In addition, another field at a distance ∼3 μm from the interface is observed only with forward *V*_b_ = +1 V, which corresponds to the potential barrier at the lateral GB. The electric field distribution with forward *V*_b_ at the front interface, at the junction, and at the lateral GB is determined by the relative equivalent resistance for carrier transport. However, the electric field peak with reverse *V*_b_ mainly locates the p–n junction (Figure S2b), which is observed inside the grain adjacent to the interface (Figure 4b). Different electric field profiles were observed in area 2 (Figure 4c), and a broad field extension was observed under reverse *V*_bs_, in contrast to the high-P device with a sharp field decay in area 2, indicating a low p-type region. The combination of areas 1 and 2 gives a broad field extension (Figure 4a). A strong field under forward *V*_b_ = +1 V in both area 1 and area 2 was observed at the lateral GB, similar to the high-P device but with a larger degree, indicating a larger potential barrier for carrier transport in this low-P device. This lateral GB moves from a location away from the interface in area 1 toward the interface in area 2 (Figures 3 and 4). These potential features and nonuniformities illustrate a more nonuniform doping concentration and mixed doping polarity in comparison to the high-P device.

**Figure 5:**
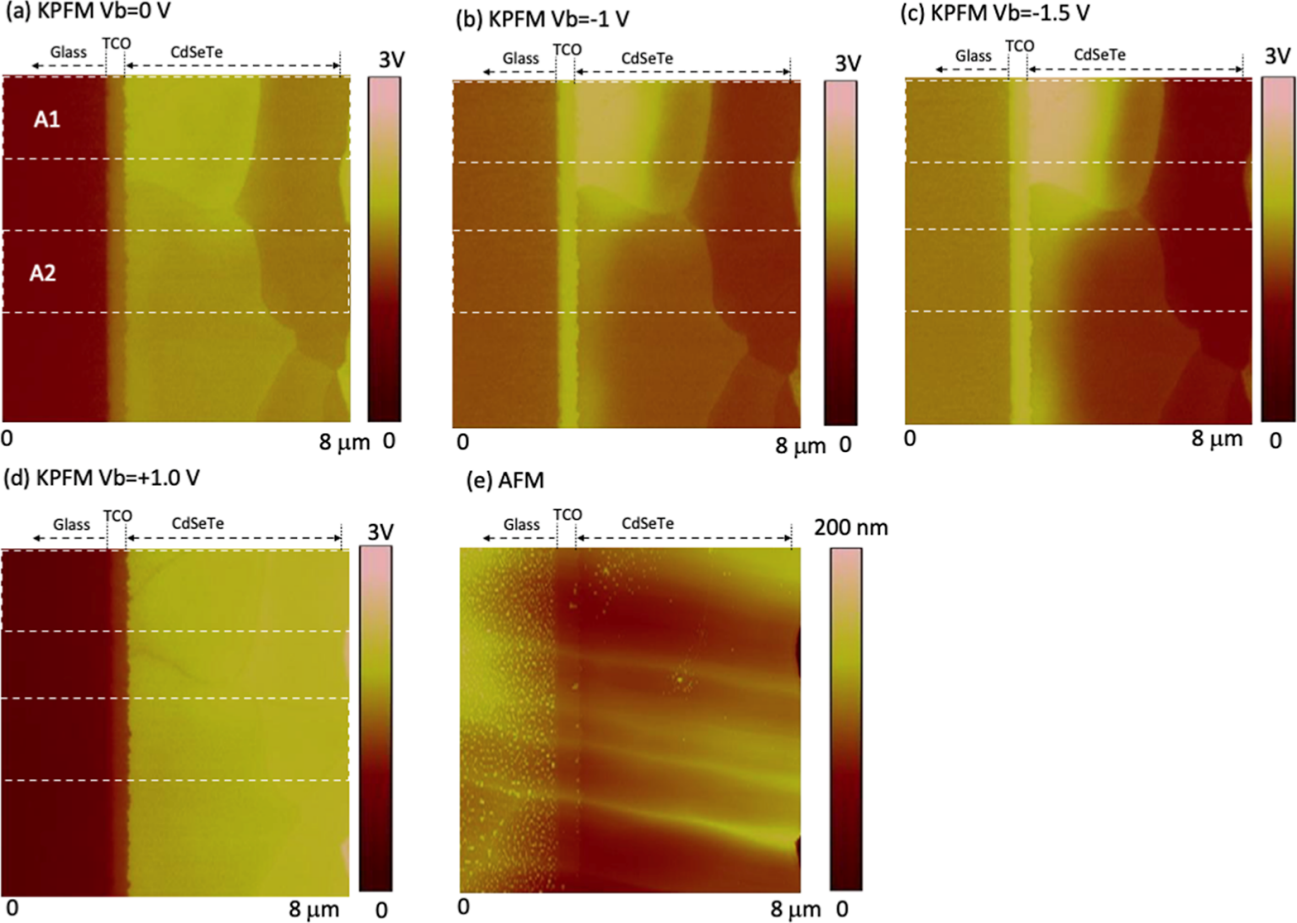
(a–d) KPFM images taken on the cross section of the no P device on the same area under different bias voltages *V*_bs_ and (e) corresponding AFM image taken simultaneously with the KPFM images. Areas 1 and 2 denote regions with different potential distributions, profiles of which are shown in Figure 6.

**Figure 6:**
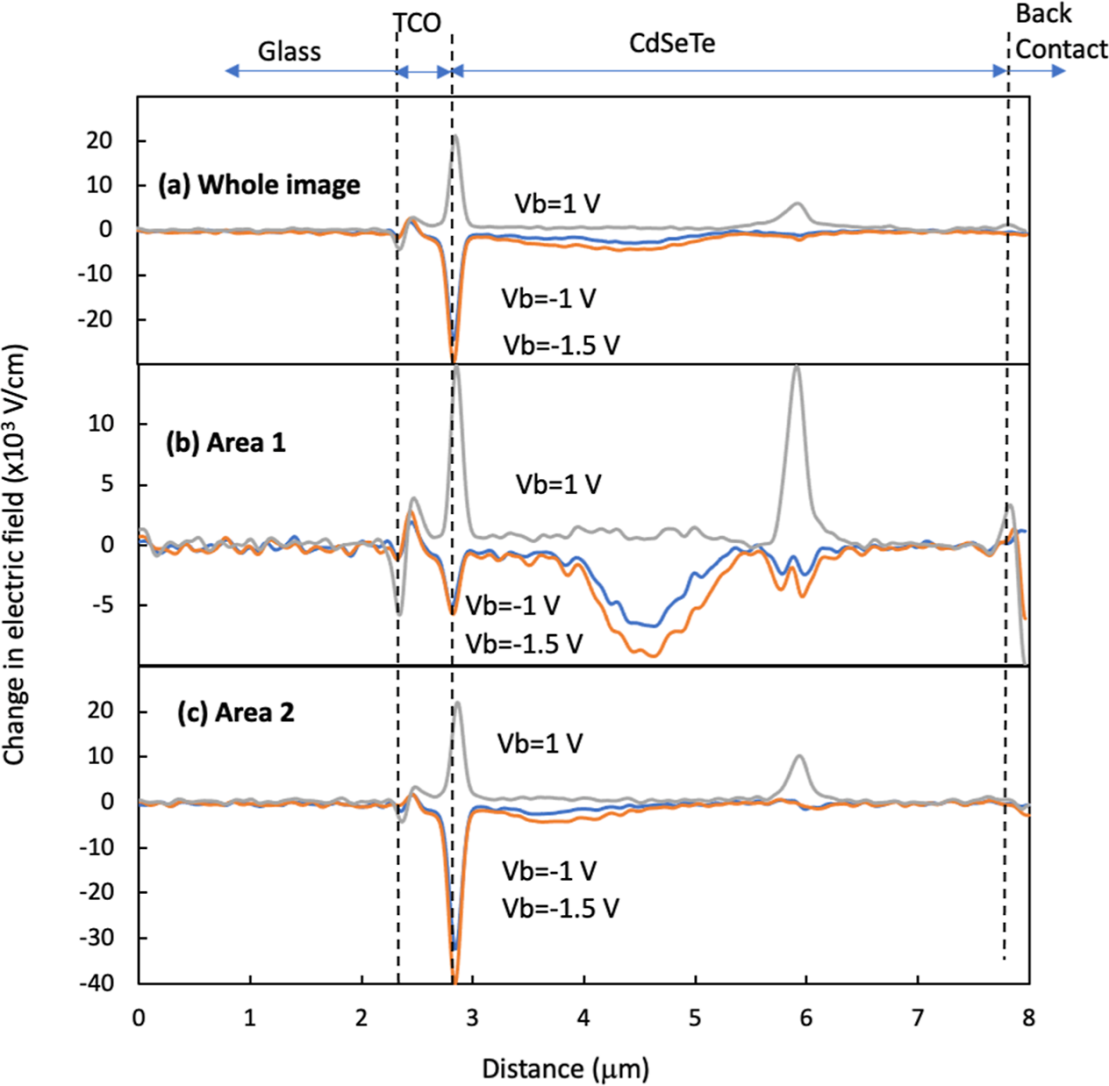
Change in the electric field induced by *V*_b_ across the no P device, averaged over (a) whole image, (b) area 1, and (c) area 2, as indicated in Figure 5.

Figures 5 and 6 show the potential images and profiles of the *V*_b_-induced electric field taken on the device without intentional doping (no P). The profiles of AFM, potential, and potential changes, together with the electric field changes, are shown in Figure S4. The potential appears much more nonuniform than that in both high-P and low-P devices. The voltage drop under reverse *V*_b_ across the absorber in the upper part of the image (area 1) appears mainly in regions around the middle depth of the absorber inside of the grain adjacent to the front interface, whereas the voltage drop across the lower grain (area 2) is around the front interface. This large location difference and the large difference in potential drop degree indicate a very large doping nonuniformity and the associated nonuniform built-in potential across the device. In other words, if the doping and built-in potential are nonuniform, the *V*_b_-induced voltage drop and electric field change are also nonuniform. Similar to the high-P and low-P devices, there are lateral GBs parallel to the device but with much more profound *V*_b_-induced electric field across the GBs under forward *V*_b_ than the P-doped devices, illustrating that P-doping effectively suppressed the potential barrier for carrier transport. The strongest field peak in area 1 under reverse *V*_b_ shifts to a distance much deep into the bulk (∼1.75 μm) with a smaller field at the front interface (Figure 6b), demonstrating the n–p junction deep inside the absorber. In area 2, although the strongest field peak was observed at the front interface, a broad peak centered ∼1 μm away from the interface was observed (Figure 6c). While detailed defect chemistry is unknown within this characterization, the film structure and Se distribution across the device and among the grains can play a significant role in defect chemistry and the associated doping polarity and concentration in this device without Gr-V doping.^8^ Correlative characterizations by techniques such as electron back scattering diffraction and scanning electron microscopy–energy dispersion spectroscopy (SEM–EDS) might be able to provide more insights on the structural and chemical causes of the electronic properties. McGott et al. reported increased n-type doping with Se content in uniformly Se-alloyed CdSeTe without intentional doping.^19^ In addition, the field at the lateral GB under forward *V*_b_ (Figure 6b,c) is much stronger than that of the P-doped devices, indicating a larger barrier for charge transport. Overall, the potential and field distributions are much more nonuniform than those of the P-doped devices, indicating a sophisticated junction structure, consistent with the inferior device performance (Table 1). These, as corroborated with the SSRM measurement discussed later, suggest a direction in reducing and ultimately eliminating the nonuniformity toward a uniform and sharp p–n junction structure at the interface.

### 2.2 SSRM Resistance Imaging

To obtain a more comprehensive picture of electrical nonuniformity, we combine the nonuniform potential drop measured by KPFM with the nanometer-scale electrical probe of SSRM for resistance mapping. The resistance images can be further extended to active charge carrier mapping by assuming a reasonable constant charge mobility, as the mobility usually varies by less degrees (limited to 1 order of magnitude^20^) than doping nonuniformity (can vary by orders of magnitude) in polycrystalline CdSeTe. This doping nonuniformity is closely associated with the potential nonuniformity measured by KPFM. The devices were delaminated at the TCO/CdSeTe interface to perform SSRM imaging on the CdSeTe absorber side. SSRM sensing volume is a hemisphere beneath the probe with a radius of ∼50 nm (Figure S5).^16,21^ We have calibrated the resistance measurement by measuring on molecular beam epitaxy-grown single-crystalline CdTe films with well-defined hole concentrations and mobility.^21^ CdSeTe was further beveled into the film bulk from the delaminated CdSeTe surface using glancing angle ion-milling, to assess the film doping at different depths from the interface (Figure S6). The film with the vertical depth of ∼5 μm was largely exposed in the lateral ∼5 mm range; each 15 μm × 15 μm scanning is within approximately the same depth, and moving the imaging area in mm order can assess the film at different depths.

**Figure 7:**
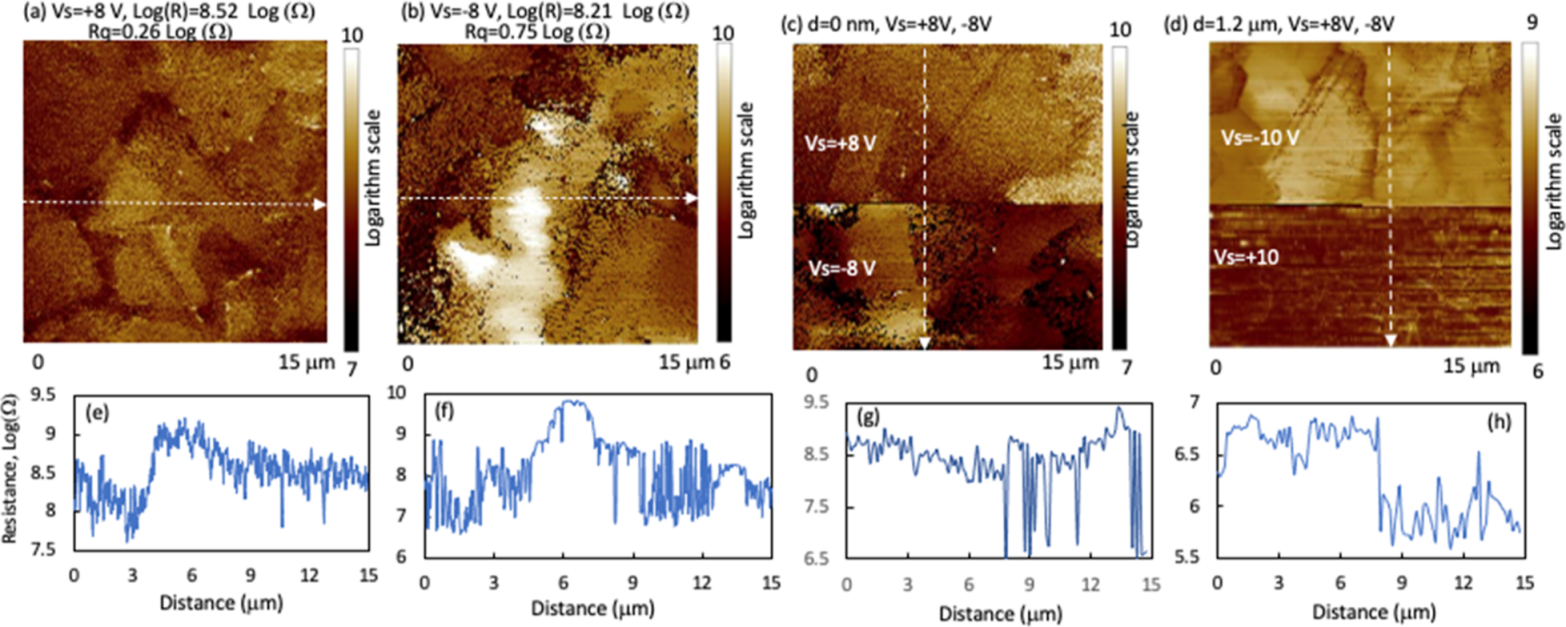
SSRM images taken on the high-P device with (a) positive, (b) negative, and (c) both *V*_s_ polarities at the delaminated interface on the CdSeTe side. (d) Image taken at a depth of 1.2 μm away from the interface with both *V*_s_ polarities. (e–h) Example resistance line profiles along the line in the corresponding images.

**Figure 8:**
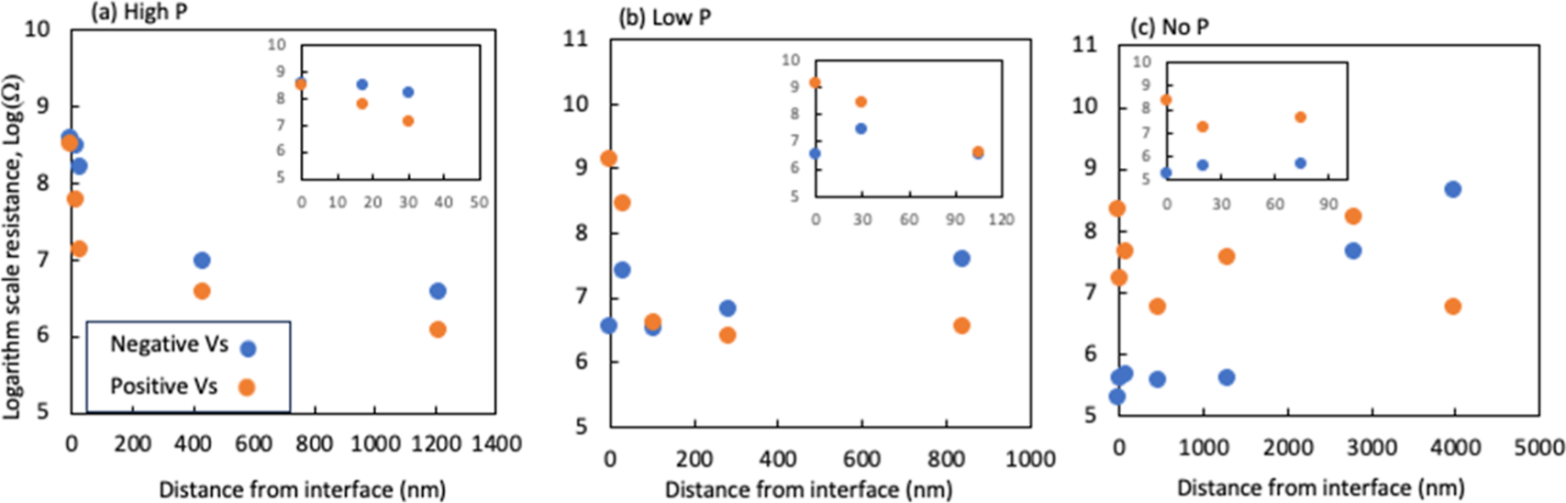
SSRM average resistance as a function of beveling depth from the interface on the three devices: (a) high-P, (b) low-P, and (c) no P. Insets show resistance at shallow depths near the interface.

Figure 7 shows SSRM resistance images on the delaminated interface of the high-P device and on further beveled surfaces down to the CdSeTe bulk. First, the average resistance values under positive (Figure 7a) and negative (Figure 7b) sample bias voltages *V*_s_ are similar, with a high resistance value of ∼10^8^ Ω. This high resistance value corresponds to a hole concentration of ∼5 × 10^14^/cm^3^, assuming a hole mobility of ∼5 cm^2^/(*V*_s_).^20^ The probe/sample contact radius of ∼22 nm, which was used for converting the spreading resistance to material resistivity, was calibrated using a crystalline CdTe thin film.^21^ In SSRM, dominating p- or n-doping polarity can be inferred by comparing the resistance values under forward or reverse bias voltages of the probe/sample contact. SSRM measures the total resistance along the current path from the probe to back contact (Figure S5). Back contact resistance is very small compared to the sample’s spreading resistance, not a comparable contributing factor, because of the large contact area. The front contact resistance is minimized sufficiently by pressing the probe into the sample and applying a large forward sample/probe bias voltage (*V*_s_). Therefore, the measured total resistance is dominated by the sample’s spreading resistance, and the spreading resistance is further dominated by the local resistivity beneath the probe as the spreading resistance contribution decreases rapidly with the distance away from the probe due to the rapid increase of conduction routes.^15,16,21^ Similar average resistance values under forward (positive) and reverse (negative) *V*_s_ (Figure 7a–c) suggest a p-type weighted doping polarity at the interface, considering the mobility of holes is lower than that of electrons.^22,23^ These resistance values and the resistances over the film depth are listed in Figure 8. However, the resistance difference between the forward and reverse *V*_s_ in high-resistance or low-doped materials can decrease due to the decrease in the barrier height difference, and spreading resistance can dominate in both *V*_s_ polarities. In other words, the doping polarity may not be readily distinguished by switching the *V*_s_ polarity for low-doped materials.

In addition to the large values, the resistance varies more than 1 order of magnitude in μm scales (Figure 7), indicating a similar active doping nonuniformity. The nonuniform doping may correlate with the doping defect and low activation rate. As the doping is p-type weighted, the resistance image under a positive *V*_s_ (Figure 7a) reflects the doping nonuniformity, and the image under reverse *V*_s_ is dominated by contact resistance. There seems to be a weak resistance contrast between GB and GI with a slightly lower resistance at the GBs. However, resistance variation between the gains is rather observed. Oxygen diffusion into the absorber from the TCO during the device processing can cause high and nonuniform resistance near the front interface.^11,24^ Gr-V dopant accumulation around the interface can induce defects and high doping compensation in the region.^2,25−27^ The interactions among the various existing chemical elements such as O, P, and Se in the CdTe zinc blende lattice can make the device deviate from the model device with abrupt front interface both metallurgically and electronically, which can complicate the local doping and electronic structure and result in the high resistance around the interface.

With beveling into the CdSeTe bulk from the delaminated surface, the resistance decreased rapidly and reached a low value of 10^7^ Ω at a depth of ∼30 nm (inside of Figure 8a) that corresponds to a carrier concentration in high 10^15^/cm^3^ range. The resistance further decreased to 10^6^ Ω at a depth of ∼400 nm corresponding to a carrier concentration of 10^16^/cm^3^. The resistance with positive *V*_s_ decreased more rapidly than that with negative *V*_s_ (Figure 8a), demonstrating the absorber transitioning to more p-type-weighted. Resistance imaging at a depth of 1.2 μm with both *V*_s_ polarities on the same image is shown in Figure 7d, exhibiting a much larger resistance with negative *V*_s_ than that with positive *V*_s_. Individual images with single *V*_s_ polarity and the corresponding AFM images are shown in Figure S7. The thin high-resistance region within ∼30 nm from the interface (Figure 8a) cannot be resolved by KPFM due to inadequate spatial resolution, but the nonuniformity in a wider depth is consistent with the small odd features showing potential drops and electric field extensions observed by KPFM.

In another note, the SSRM data on the delaminated CdSeTe surface appear “noisy”, showing oscillation at a submicron length scale in addition to the nonuniformity of resistance variation at the scale of several microns (Figure 7) because the delaminated surface has a surface corrugation of ∼50 nm in the lateral sub-μm scale (Figure S8), which follows the corrugation of the TCO/CdSeTe interface and makes the probe/sample contact area oscillate in the SSRM imaging. This “noise” decreased significantly with bevel-milling into the film bulk (Figure 7), as the surface became smoother by the ion-milling in the glancing angle.

**Figure 9:**
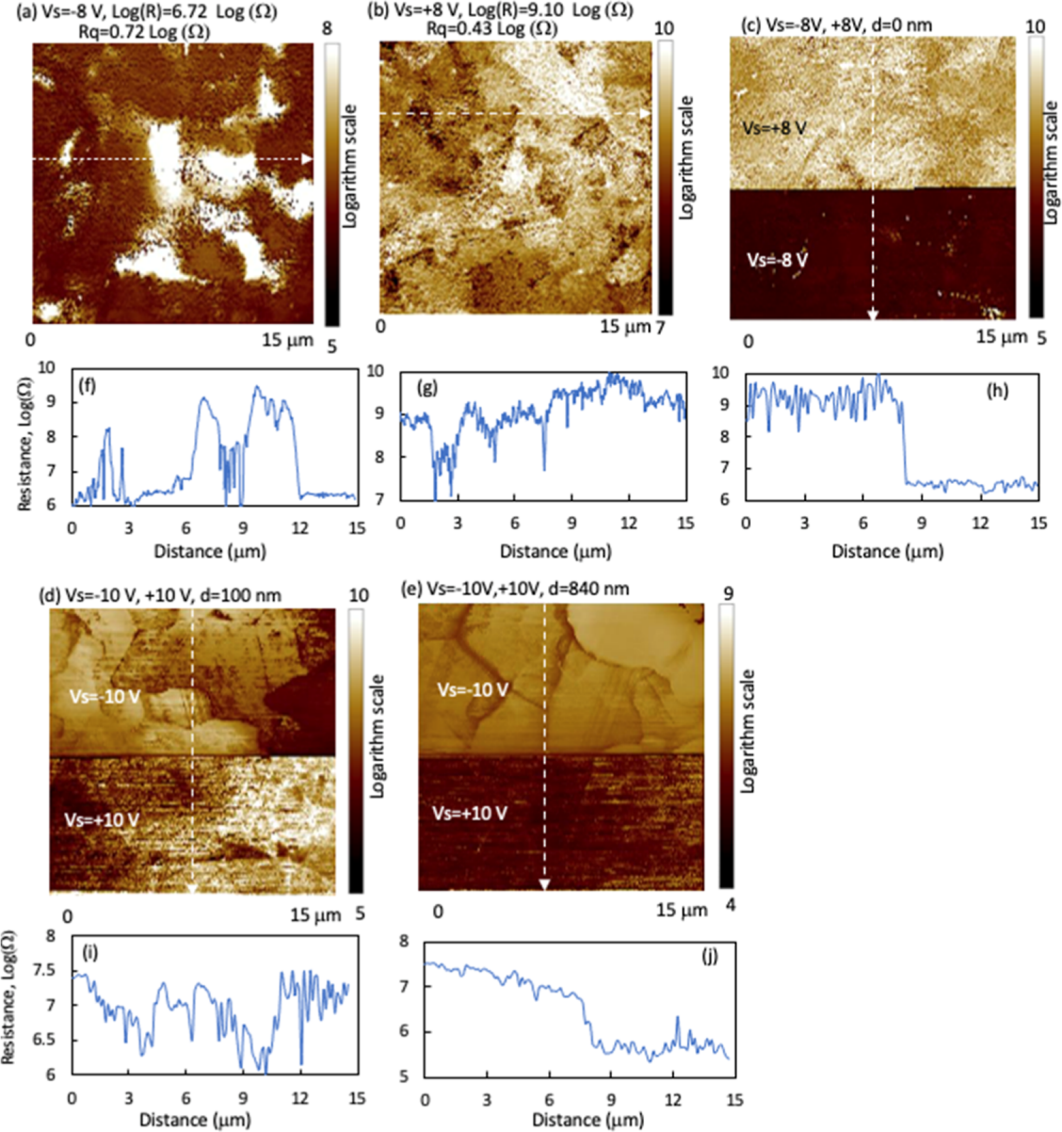
SSRM images taken on a low-P device with (a) negative, (b) positive, and (c) both *V*_s_ polarities at the delaminated interface on the CdSeTe side. (d,e) Images taken at depths of 100 and 840 nm away from the interface with both *V*_s_ polarities. (f–j) Example resistance line profiles along the dashed line in the corresponding images.

Figure 9 shows SSRM images taken on the low-P device, on the delaminated surface, and with beveling into the bulk. Different from the high-P device, the resistance with negative *V*_s_ is more than 2 orders of magnitude lower than that with positive *V*_s_ (Figure 9a–c), illustrating that the absorber at the interface is overall n-type with an electron concentration of 3 × 10^15^/cm^3^ (assuming electron mobility of ∼50 cm^2^/(*V*_s_), 1 order of magnitude larger than hole mobility^22,23^). The electron density varies largely by 2–3 orders of magnitude at the interface on several μm scales, but no clear resistance contrast at the GBs was observed.

With beveling into the film bulk, the resistance under positive *V*_s_ decreased rapidly, while the resistance under negative *V*_s_ did not change significantly (Figure 8b). At a depth *d* ∼100 nm from the interface, the average resistance values under positive and negative Vs are about balanced (Figures 8b and 9d), indicating a slightly p-type weighted doping, considering a smaller hole mobility than electron. The average resistance value suggests a hole concentration of 3 × 10^16^/cm^3^, by assuming a hole mobility of 5 cm^2^/*V*_s_.^20^ The nonuniformity of resistance (also carrier concentration) decreased at this depth compared to the interface, fluctuating by about 1 order of magnitude. SSRM images at this depth with single *V*_s_ are shown in Figure S9.

With further beveling to a depth *d* ∼840 nm, the resistance with positive bias decreased slightly; however, the resistance with negative Vs increased significantly (Figures 8b and 9e), illustrating a further transition to more p-type weighted. SSRM images at this *d* ∼840 nm with single *V*_s_ are shown in Figure S10. The overall resistance transition with depth is consistent with the KPFM potential drop on the cross section, where a peak of the potential drop was observed at *d* ∼750 nm away from the interface in some areas, indicating a buried junction at that depth, although broad field extensions were detected around the interface in some other areas that indicated low doping. We note that the nonuniform doping and potential drops are different area by area; therefore, we cannot draw a fully consistent combination of doping and potential maps without imaging on the same area. Besides, the potential was imaged on the cross section and SSRM on the planar view. However, the general results of potential drop at locations hundreds of nanometers away from the interface and the overall n–p transition at ∼100 nm depth consistently indicate a nonuniform microelectronic structure in the region near the front interface.

**Figure 10:**
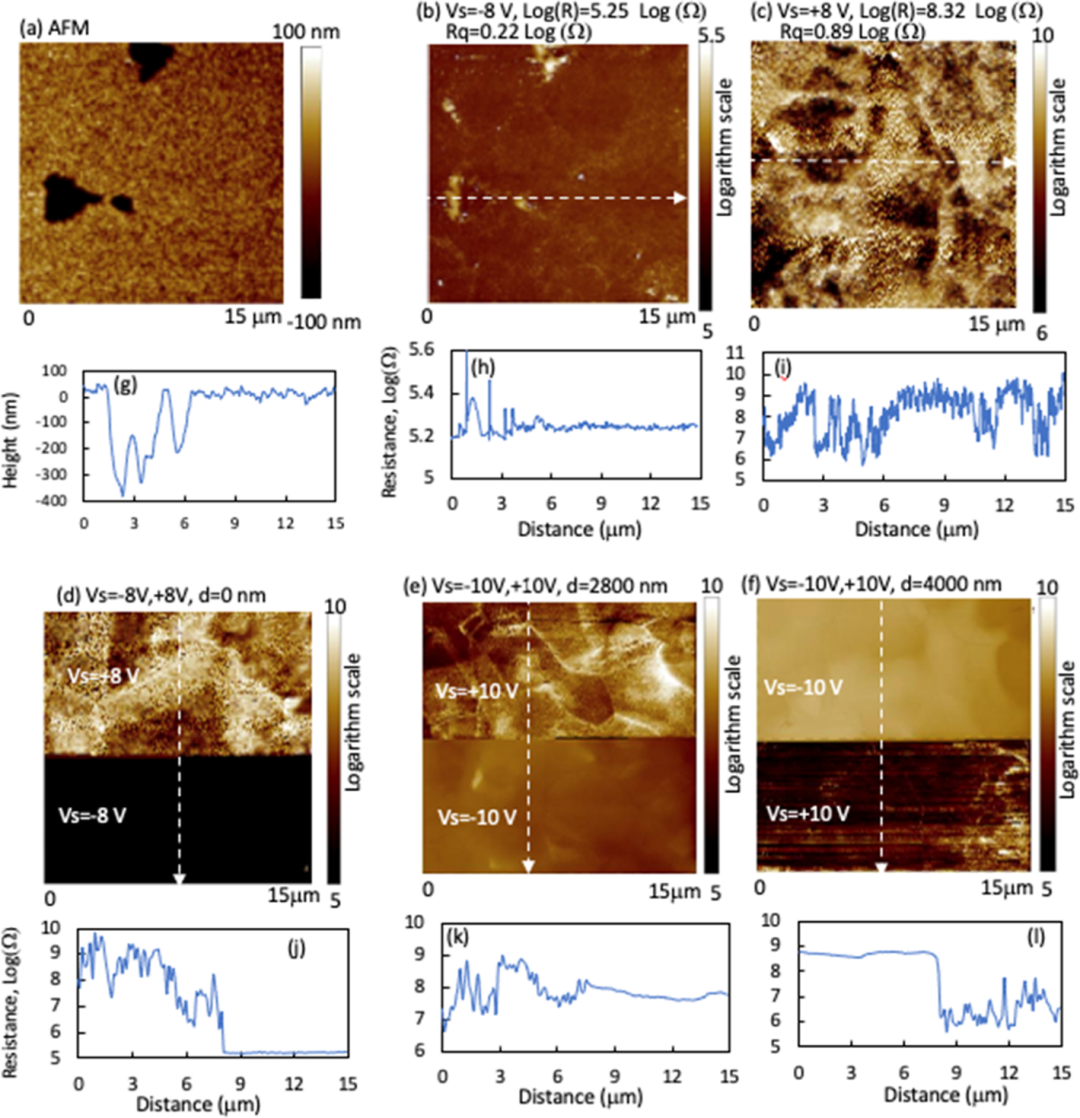
SSRM images taken on the no P device with (b) negative, (c) positive, and (d) both *V*_s_ polarities at the delaminated interface on the CdSeTe side. (a) Corresponding AFM image to the SSRM image (b), showing voids at the interface. (e,f) Images taken at depths of 2.8 and 4 μm away from the interface with both *V*_s_ polarities. (g–l) Line profiles along the line in the corresponding images (a–f).

Figure 10 shows SSRM images taken at different depths of the no P device. The resistance at the interface with negative *V*_s_ is more than 3 orders of magnitude smaller than that of positive *V*_s_ (Figures 8c and 10b,c), demonstrating n-type doping at the interface. The resistance is low, corresponding to 9 × 10^16^/cm^3^ electron density by assuming an electron mobility of 50 cm^2^/*V*_s_. The resistance is rather uniform (Figure 10b), different from the P-doped devices, except for some regions with voids as shown in the AFM image (Figure 10a). A very weak resistance contrast at the GBs was observed, with a slightly higher resistance at the GBs of the n-type region. The overall resistance with negative *V*_s_ increases with depth and is about balanced with positive *V*_s_ at a deep depth of ∼2.8 μm from the interface (Figures 8c and 10e). Individual SSRM images with single *V*_s_ at this beveling depth are shown in Figure S11. Grain structure is shown in the image with positive *V*_s_, with a higher resistance on the GBs. A large resistance contrast of more than 1 order of magnitude between grains is also observed. We note that, overall, the resistance contrast at the GBs is not or very weakly observed in all of the depths of the three devices, except for this n–p transition depth of the no P film, the mechanism of which is unknown. The fact that no clear GB contrast was observed indicates no significant contribution of GB scattering to carrier mobilities. The carrier depletion or accumulation by the electric field around the GBs is not measured by SSRM, since the large forward *V*_s_ between the probe and sample flattens out the electrical potential around the GB.

With further beveling toward the device backside, the resistance with positive Vs decreases, corresponding to a hole concentration of 3 × 10^16^/cm^3^ (mobility of 5 cm^2^/*V*_s_) at a depth of 4 μm (Figures 8c, 10f and S12). The resistance varies significantly by more than 1 order of magnitude but does not clearly follow the grain structure (Figure S12a). The n–p transition at the deep depth, as revealed by the resistance imaging, is consistent with the local deeply buried n–p junction imaged by KPFM. In this CdSeTe absorber without any intentional doping, intrinsic defect doping plays a dominant role. The relatively heavy n- and p-doping in the front and back regions of the device illustrate varying defect chemistry, atomic structure, and subsequently electronic structures across the device. The device has a Se profile weighted more on the front side that can significantly change the defect chemistry. McGott el al. reported that more n-doping was found in devices with more Se incorporation in uniformly Se-alloyed devices.^19^ We observed that the lateral nonuniform resistance distribution is associated with Se nonuniformity. Beside Se alloying, CdCl_2_ post anneal can be a driver for the large doping contrast between the film’s front and back sides, as Cl diffuses from the back to front sides.^7,28^ Defects of cation–anion antibonding and anion vacancies can be a source for n-doping.^29^ The voids observed at the interface of the no P device (Figure 10a) may result from excessive Se diffusion from the front side toward the film bulk during CdCl_2_ annealing, suggesting more anion vacancy formation near the front interface. On the other hand, the high p-type doping at the back side may result from Cd vacancy, other acceptor defects, or defect complexes.^29^ Overall, the uncontrolled intrinsic defect doping in the no P device may result in the largest nonuniform doping and potential drop among the three devices. With the progressive P-doping, the CdSeTe absorber changed to more p-type weighted in the region near the front interface, and the device performance was largely improved (Table 1).

## 3 Conclusions

We investigated the microelectronic structure of CdSeTe devices with three levels of P-doping. The KPFM potential and SSRM resistance imaging corroborate each other and illustrate a nonuniform doping at various depth ranges of the devices, which is associated with a nonuniform potential drop across the devices. These microelectronic structures were improved by enhancing P-doping. The no P device exhibits an overall n–p transition deep inside CdSeTe and a large potential nonuniformity across the device. With P-doping, the overall n–p transition depth of the low-P device decreased to the ∼100 nm range, and the nonuniform potential drop is mostly confined around the front interface. With increasing P-doping to high P, doping at the front interface and nearby is weighted to p-type, and the odd potential drop is observed scarcely in small areas. These characterizations point to the direction of eliminating the nonuniform doping and irregular potentials for improving the device efficiency, in addition to more common strategies such as absorber lifetime, band alignment, recombination at the front and back interfaces, and so on.

## 4 Methods

KPFM is based on noncontact mode AFM (Veeco Dimension 3100 and Nanoscope V controller) and is homemade using the second harmonic oscillation of cantilever to enhance voltage sensitivity in ∼10 mV and to isolate from the artifact of surface morphology.^13^ A PtIr-coated Si probe (Nanosenser PPP-EFM) with a resonant oscillation frequency of ∼60 kHz was used. The first harmonic oscillation is used for AFM imaging. TCO is grounded, and a bias voltage *V*_b_ is applied to the back contact to vary the voltage drop across the device. The device was mechanically cleaved to expose cross sections, and the cross section was polished by Ar-ion-milling (JEOL CP). The sample was further annealed in a low-vacuum oven at 250 °C for 5 min to passivate the cross-sectional surface.^17^ In these sample preparation steps, a few and randomly distributed weak local shunting points may occur due to mechanical stress on the device by cleaving, which can make slight changes in *I*–*V* of cleaved device pieces. However, these moderate processing conditions do not change the device’s chemical, structural, and electronic properties, except for the weak local shunting point. In fact, KPFM images were taken on multiple local micrometer regions, and the images show consistent results on randomly selected regions, as the imaged area on cross sections is away from the random weak shunting points.

SSRM is based on the contact mode of AFM (Bruker Dimension Icon and Nanoscope V) and set in an Ar-glovebox with a logarithm-scale amplifier (Bruker SSRM module) to measure a wide range of resistance (10^3^–10^14^ Ω). The probe (Bruker DDESP-V2) is a highly doped diamond-coated Si tip. SSRM is a two-terminal resistance measurement with a bias voltage applied to the sample (*V*_s_), and the probe is floating-grounded. The back contact resistance along the current route, *R*_b_, is very small (10^2^ ∼10^3^ Ω) compared to the sample’s spreading resistance *R*_sp_, which is neglectable. The Front probe/sample contact *R*_c_ is minimized by pressing the probe into the sample in ∼mN force to creating local dangling and strained bonds and applying a large forward *V*_s_ (8–10 V).^15,16,21^ The measured resistance is thus dominated by *R*_sp_, and further by the resistance beneath the probe in ∼50 nm sizes. For sample preparation, the front interface was delaminated by thermomechanical stressing in liquid nitrogen. The absorber was further beveled into absorber bulk using Ar-ion-milling (JEOL CP) at a glancing angle to make the film with μm depth appear in a wide lateral distance of mm. However, the sample was exposed to air after ion-milling during transfer to the AFM glovebox, which compromised the SSRM data quality at different depths. The beveling depth was estimated based on the measurement using an optical profilometer (Veeco WYKO NT1100).

## Supporting Information Available

Additional description of KPFM data processing and its rationality; schematics of KPFM and SSRM experimental setup, and additional KPFM and SSRM results supporting the results in the main text (PDF)
