# Supplementary material for: Microelectronic Structure and Doping Nonuniformity of Phosphorus-Doped CdSeTe Solar Cells
Source: ACS Appl Mater Interfaces. 2025 Jan 6;17(2):3278–88. doi: 10.1021/acsami.4c15741 (PMC11744667; doi:10.1021/acsami.4c15741)
Supplement: Supplementary file 1 — am4c15741_si_001.pdf [file am4c15741_si_001.pdf]

Supporting Information

Microelectronic Structure and Doping Nonuniformity of Phosphorus-Doped CdSeTe Solar Cells

Chun-Sheng Jiang[1*], Rouin Farshchi[2], Timothy Nagle[2], Dingyuan Lu[2], Gang Xiong[2], Lorelle M. Mansfield[1], and Matthew O. Reese[1]

[1]National Renewable Energy Laboratory, Golden, Colorado 80401, USA

[2]California Technology Center, First Solar Inc., Santa Clara, California 95050, USA

*Corresponding author email: chun.sheng.jiang@nrel.gov

Statement of KPFM data processing and rationality

The measurement and data processing procedure is: Taking the 2D surface potential at the different bias voltages of Vb=0, -1V, -1.5V, and +1V. We then average the potential line profiles in areas with similar potential profiles. Then the potential change in that area induced by Vb is obtained by subtracting the potential profile at Vb=0 from each at the various Vb. Finally, E field changes at X direction by Vb (across the device) was obtained by taking dV/dx. Why we do data processing in this way is because:

(a) Because the potential data are on the cross-sectional surface that are affect largely by surface charges trapped on the cross-sectional surface. To avoid the effect of surface charge and get the potential in the bulk, we need to subtract the potential at Vb=0 from each potential to obtain potential change that is approximately identical to the Vb-induced potential change in the bulk, as surface charges are localized on the surface states and do not move in a small Vb<2V

(b) We average the potential line profiles is because taking a derivative to get E field in the x direction from single potential profile would be too noisy, as any small potential change would be much enhanced by taking the derivative, so we need to average the potential profiles in areas with similar potential features, to enhance the signal/noise ratio.

(c) Since we are able to get only the potential change in the bulk by Vb, but no the built-in potential at Vb=0 and the absolute potentials at each of the Vb in the bulk, the potential change by Vb is mainly on the X direction since Vb is applied across the device.

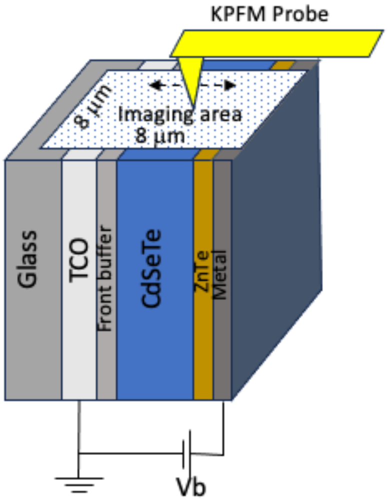

Fig. S1. A schematic showing device structure and geometry of KPFM potential imaging on cross-sections of the device.

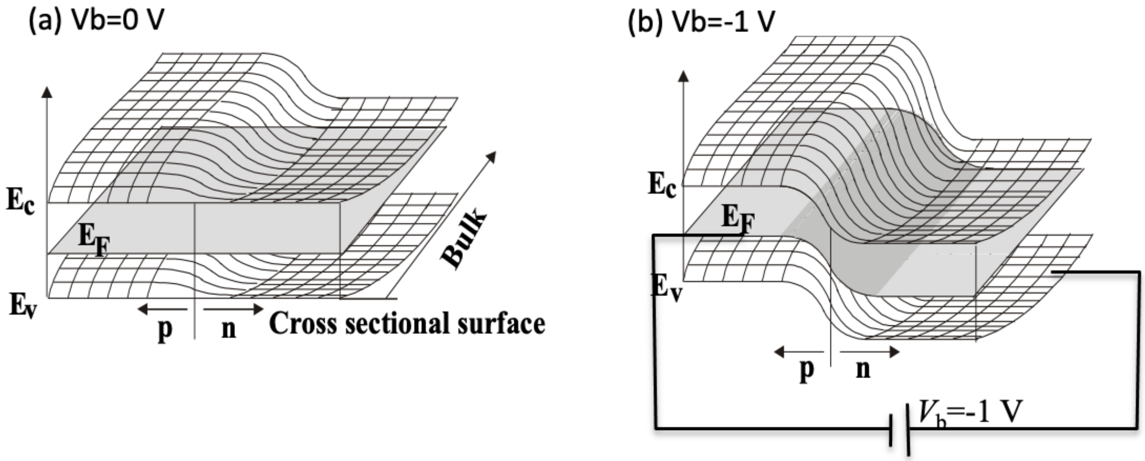

Fig. S2. A schematic showing the band diagrams with charges trapped on cross-sectional surface with (a) bias voltage Vb=0 V and (b) reverse bias of Vb=-1 V applied to the device.

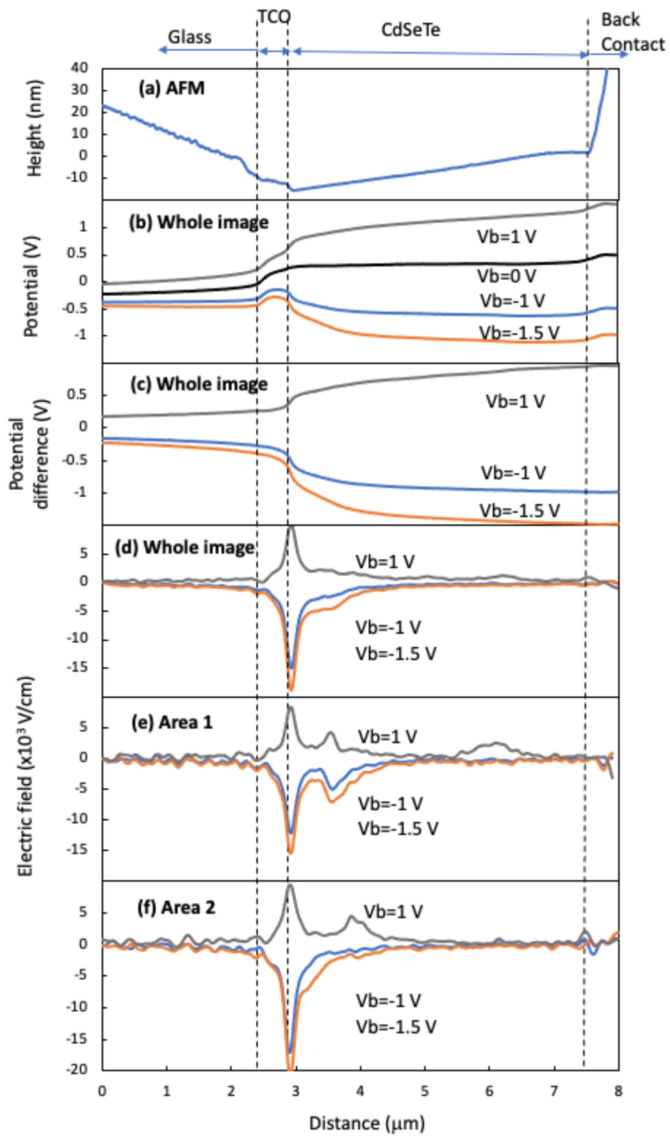

Fig. S3 (a)-(d) Profiles of AFM, potential, potential difference, and electric field difference of the LowP device, averaged from whole scan area in Fig. 3. (e)(f) are averaged electric field from the areas indicated in Fig. 3.

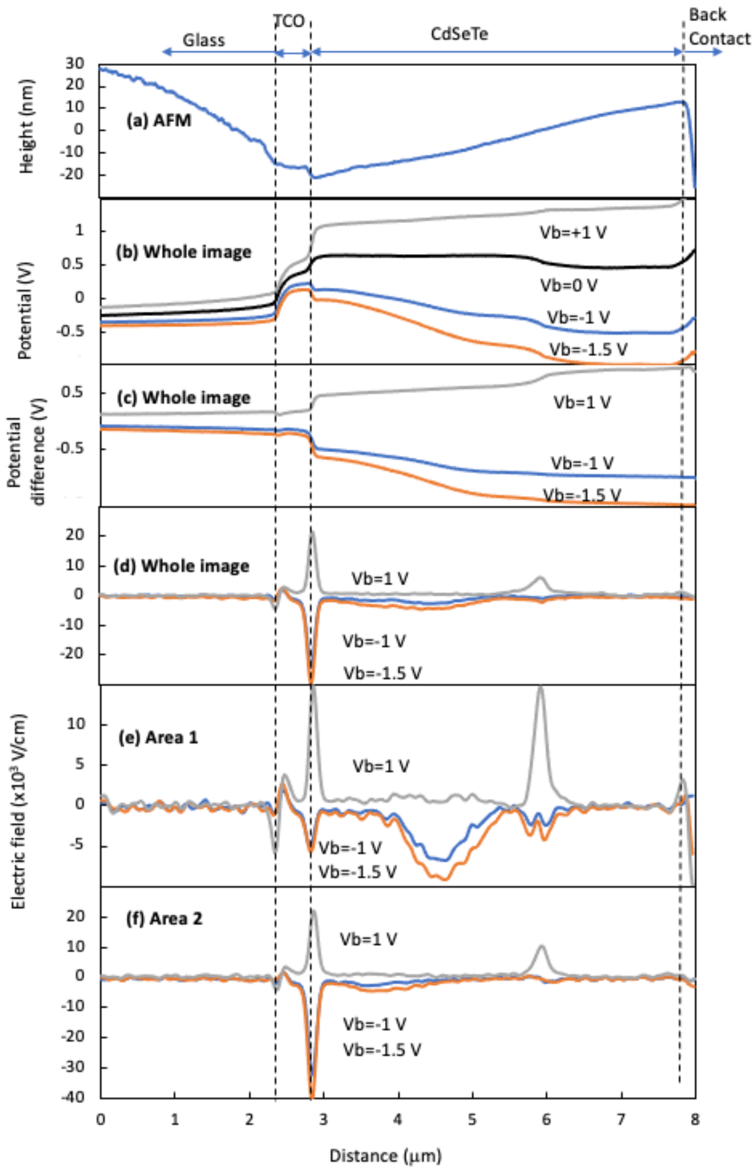

Fig. S4 (a)-(d) Profiles of AFM, potential, potential difference, and electric field difference of the NoP device, averaged from whole scan area in Fig. 5. (e)(f) are averaged electric field from the areas indicated in Fig. 5.

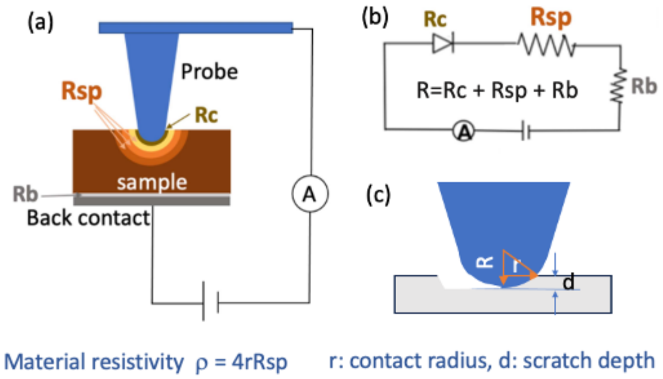

Fig. S5(a). A schematic showing SSRM set up; (b) an equivalent circuit of the SSRM measurement, and (c) a schematic of prob/sample configurations.

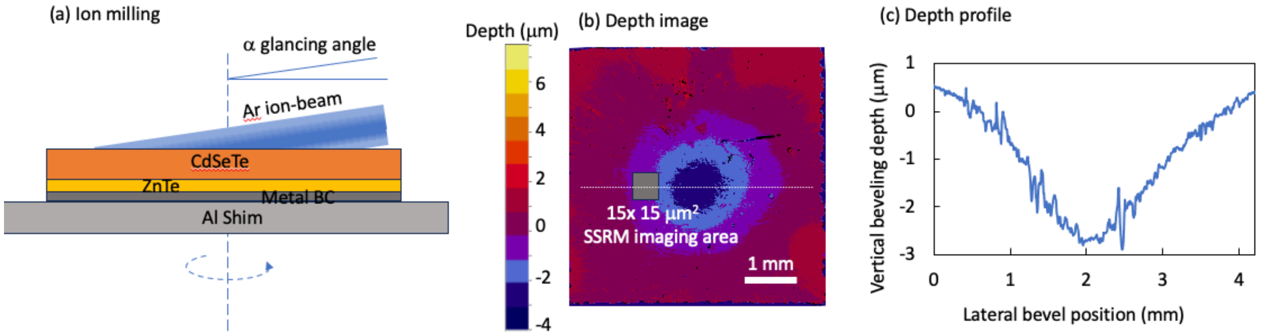

Fig. S6. (a) A schematic showing glancing-angle ion-milling beveling on the CdSeTe film delaminated from the TCO/CdSeTe interface. (b) and (c) shows the conversion of vertical depth and lateral distance from the beveling center, based on an optical profilometry measurement. A SSRM imaging area is drawn on the beveled film (not in scale).

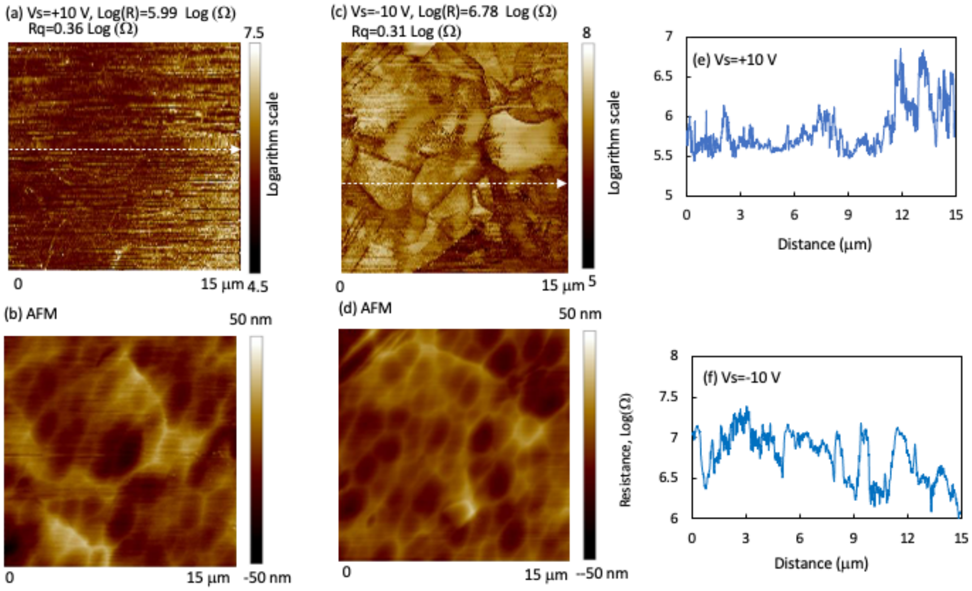

Fig. S7(a)(c) SSRM images taken on the HighP film at a depth of 1.2 µm with the forward and reverse sample bias voltages, (b)(d) the corresponding AFM images, and (e)(f) example resistance line profiles along the lines in the SSRM images.

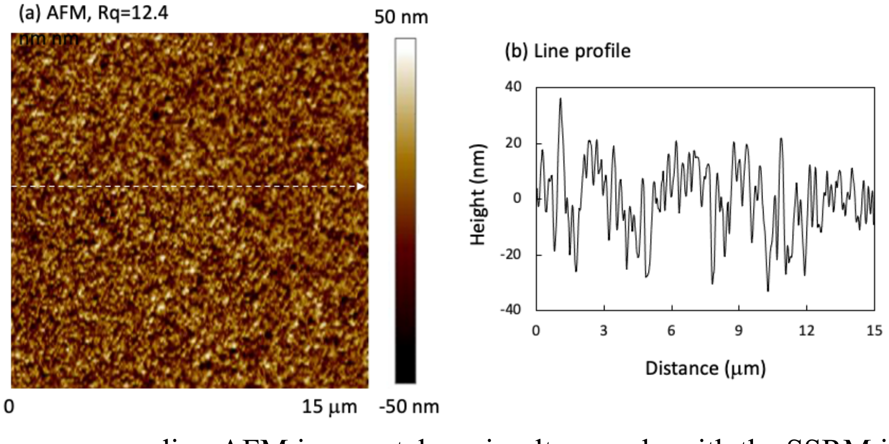

Fig. S8, (a) the corresponding AFM images taken simultaneously with the SSRM image of Fig. 7(a). (b) an AFM line profile along the dashed line in Fug. 8(a) showing the surface corrugation of ~50 nm in lateral sub-µm scale.

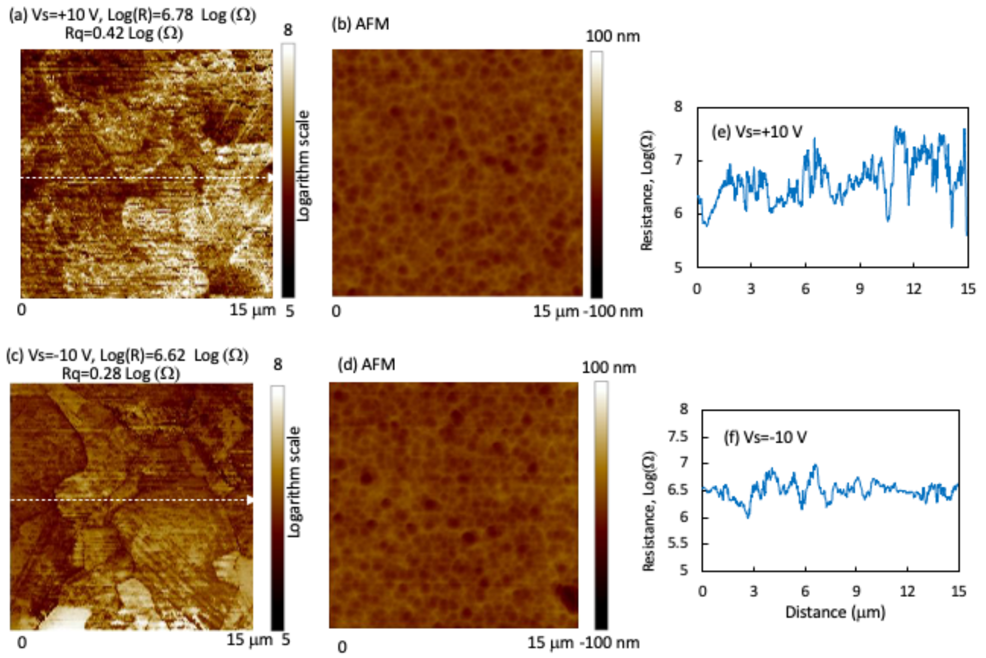

Fig. S9(a)(c) SSRM images taken on the LowP film at a depth of 100 nm, (b)(d) the corresponding AFM images, and (e)(f) example resistance line profiles along the lines in the SSRM images.

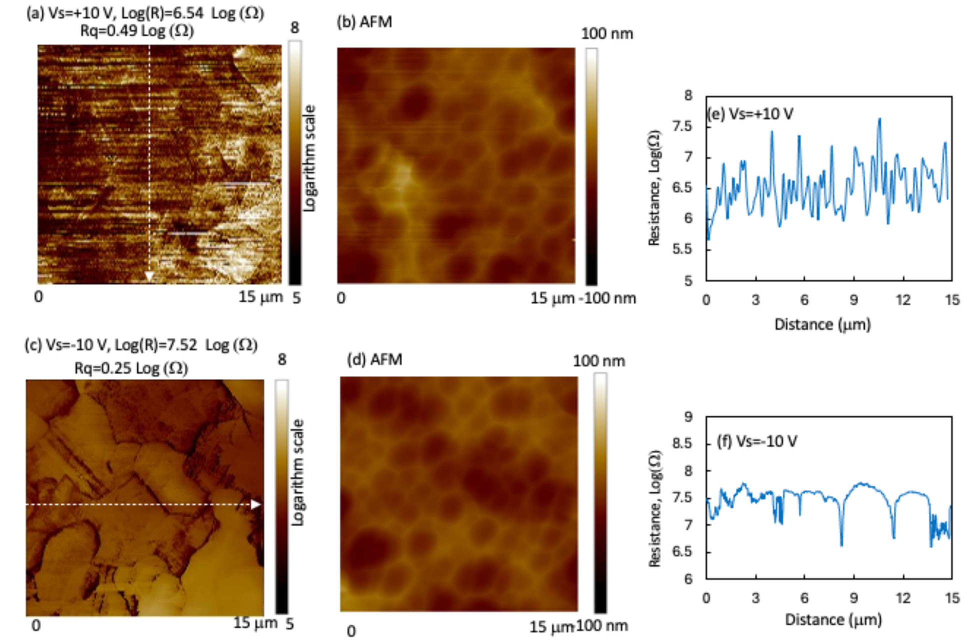

Fig. S10(a)(c) SSRM images taken on the LowP film at a depth of 840 nm, (b)(d) the corresponding AFM images, and (e)(f) example resistance line profiles along the lines in the SSRM images.

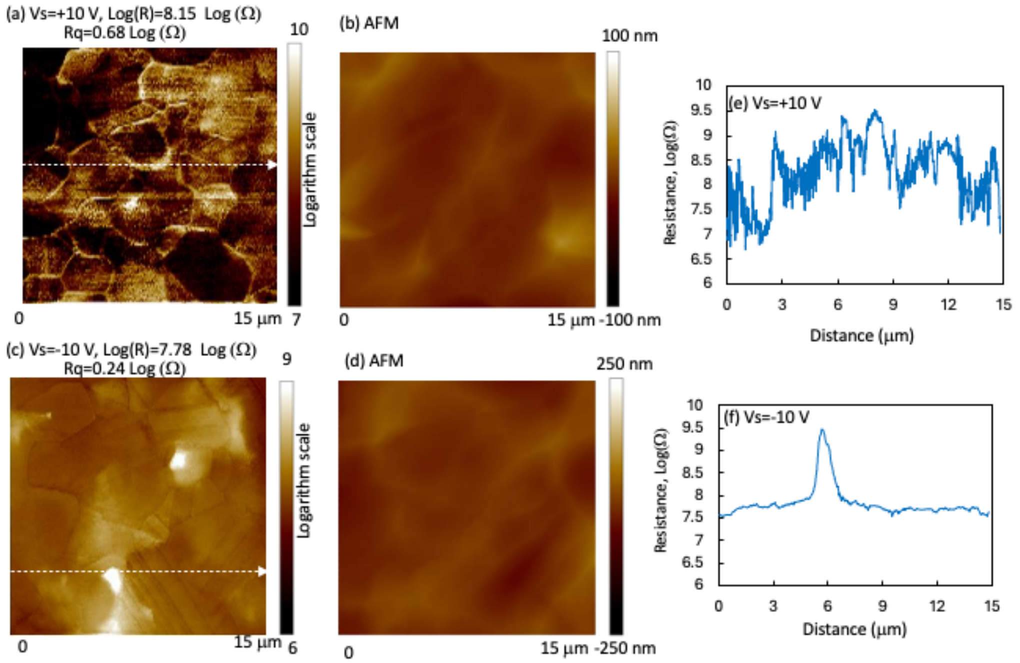

Fig. S11. (a)(c) SSRM images taken on the NoP device at a depth of ~2.8 µm away from the interface, (b)(d) the corresponding AFM images, and (e)(f) example resistance line profiles along the lines in the SSRM images.

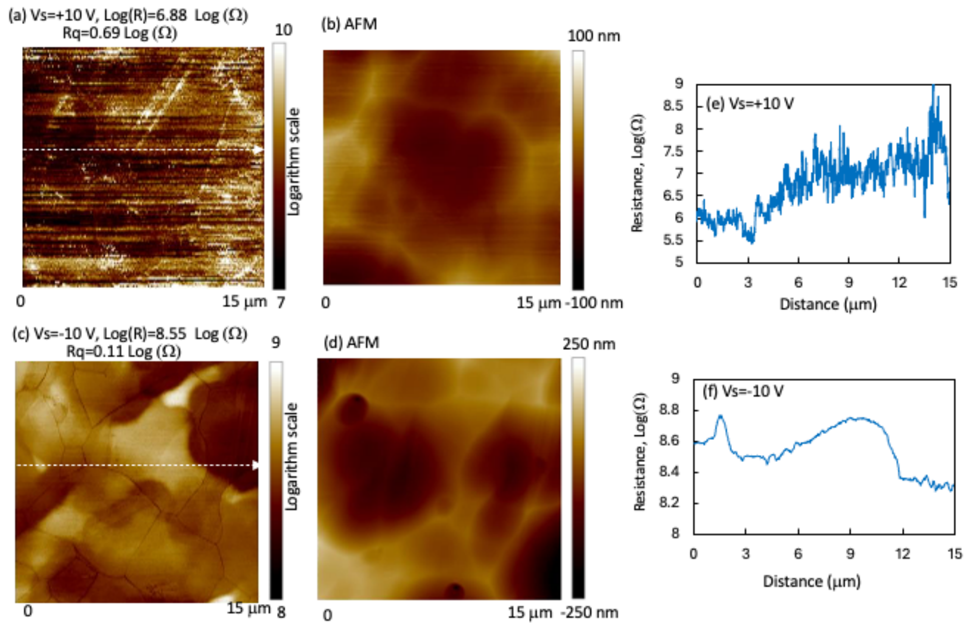

Fig. S12. (a)(c) SSRM images taken on the NoP device at a depth of ~4 µm away from the interface, (b)(d) the corresponding AFM images, and (e)(f) example resistance line profiles along the lines in the SSRM images.
